# Supplementary material for: Structure-Activity Relationships of Holothuroid’s Triterpene Glycosides and Some In Silico Insights Obtained by Molecular Dynamics Study on the Mechanisms of Their Membranolytic Action
Source: Mar Drugs. 2021 Oct 25;19(11):604. doi: 10.3390/md19110604 (PMC8625879; doi:10.3390/md19110604)
Supplement: Supplementary file 1 [file marinedrugs-19-00604-s001.zip › marinedrugs-141267 - supplementary.pdf]

Structure-Activity Relationships of Holothuroid's Triterpene Glycosides and Some In Silico Insights Obtained by Molecular Dynamics Study on the Mechanisms of their Membranolytic Action

Elena A. Zelepuga, Alexandra S. Silchenko, Sergey A. Avilov and Vladimir I. Kalinin*

Supplementary materials

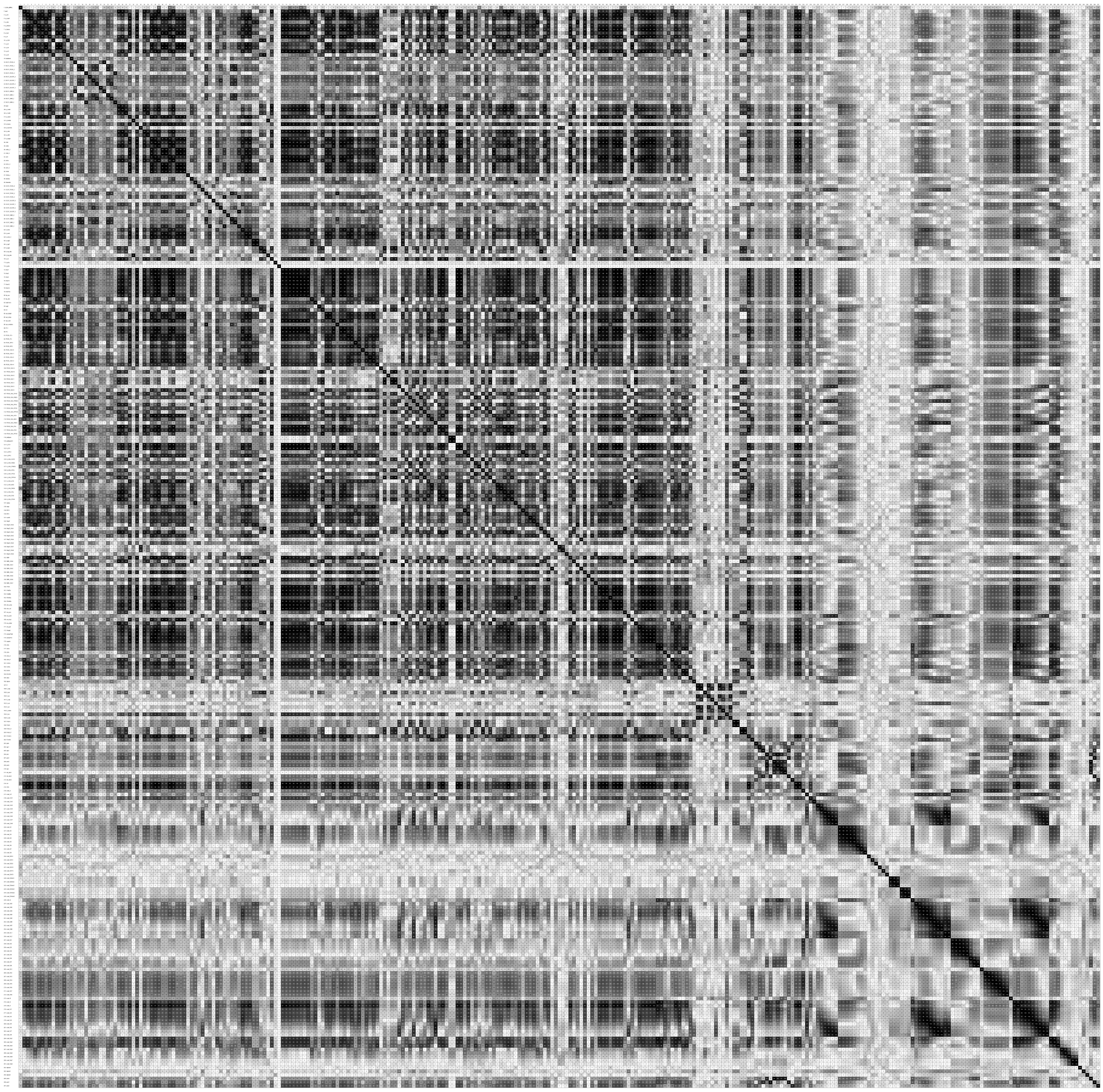

Figure S1. The Correlation matrix of the hemolytic activities of glycosides in vitro (ED50, μM/mL, Table 1) and certain calculated molecular 2D and 3D descriptors conducted with the QuaSAR-Descriptor tool of MOE 2020.0901 CCG software [1]. Moderate positive correlation of their activity with the atomic contribution to Log of the octanol/water partition coefficient (h_logP) [2], the total negative VDW surface area (Å2), the number of oxygen atoms (a_no), the atomic valence connectivity index (chi0v), kappa shape indexes (Kier) [3], describing different aspects of molecular shape, the molecular VDW volume (Vol, vdw_vol, VSA_acc, (Å3)) were disclosed.

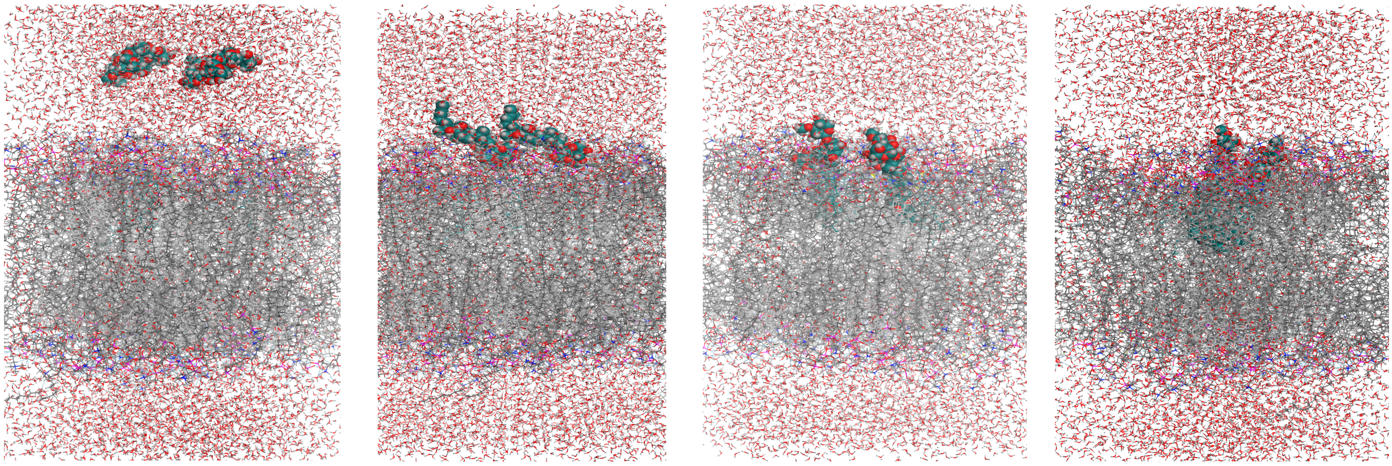

A B C D

Figure S2. (A) Initial conformation of cucumarioside A8 **(44)** for MD simulations, where the A8 **(44)** molecules are placed at a distance of 11 Å above the outer membrane leaflet with their long axis is directed along the membrane surface. **(B)** The snapshot of 85 ns MD simulations indicating the cucumarioside A8 carbohydrate parts come up to the phospholipid heads of the outer membrane leaflet. **(C)** The snapshot of 130 ns MD simulations indicating the cucumarioside A8 aglycone pass through the outer membrane leaflet. **(D)** The last snapshot of MD simulations indicating the aglycone moieties of two cucumarioside A8 molecules induce the "pore-like" complex formation inside the membrane. The glycoside is presented as cyan "ball" model, POPC+PSM +CHOL are presented as grey stick models. The solvent molecules and some membrane components are deleted for simplicity.

References

1. Molecular Operating Environment (MOE), 2019.01; Chemical Computing Group ULC, 1010 Sherbooke St. West, Suite #910, Montreal, QC, Canada, H3A 2R7,2020.

2. Wildman, S.A.; Crippen, G.M. Prediction of Physiochemical Parameters by Atomic Contributions. *J. Chem. Inf. Comput. Sci.* **1999**, 39, 868–873.

3. Hall, L.H.; Kier, L.B. The Molecular Connectivity Chi Indices and Kappa Shape Indices in Structure-Property Modeling. *Rev. Comput. Chem.***1991**, 2, 367–422. DOI: 10,1002/9780470125793,ch9
