# Supplementary figures and images for: Structure-Activity Relationships of Holothuroid’s Triterpene Glycosides and Some In Silico Insights Obtained by Molecular Dynamics Study on the Mechanisms of Their Membranolytic Action
Source: Mar Drugs. 2021 Oct 25;19(11):604. doi: 10.3390/md19110604 (PMC8625879; doi:10.3390/md19110604)

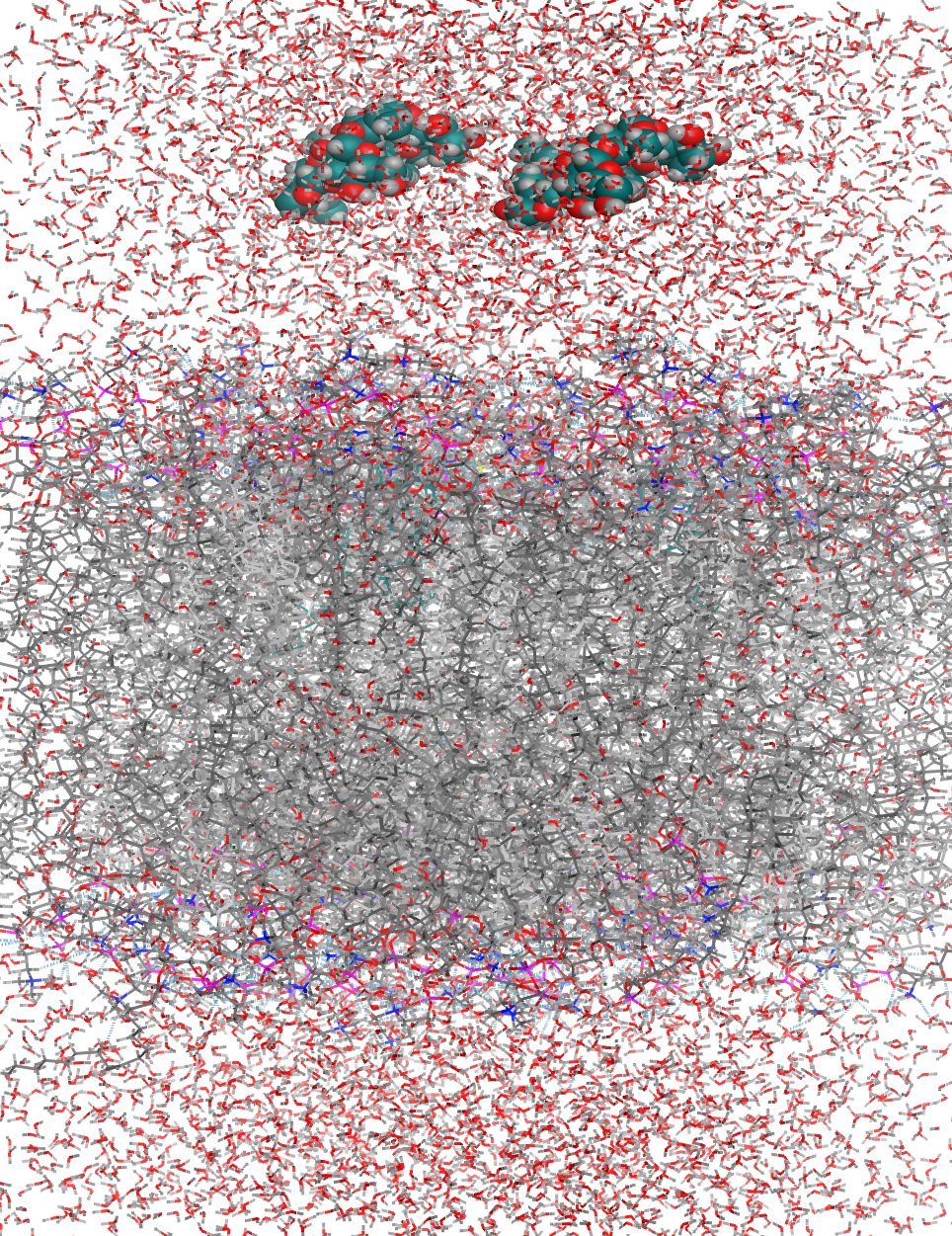

Supplement: Supplementary file 1 [file marinedrugs-19-00604-s001.zip › figure S2 A corp.jpg]

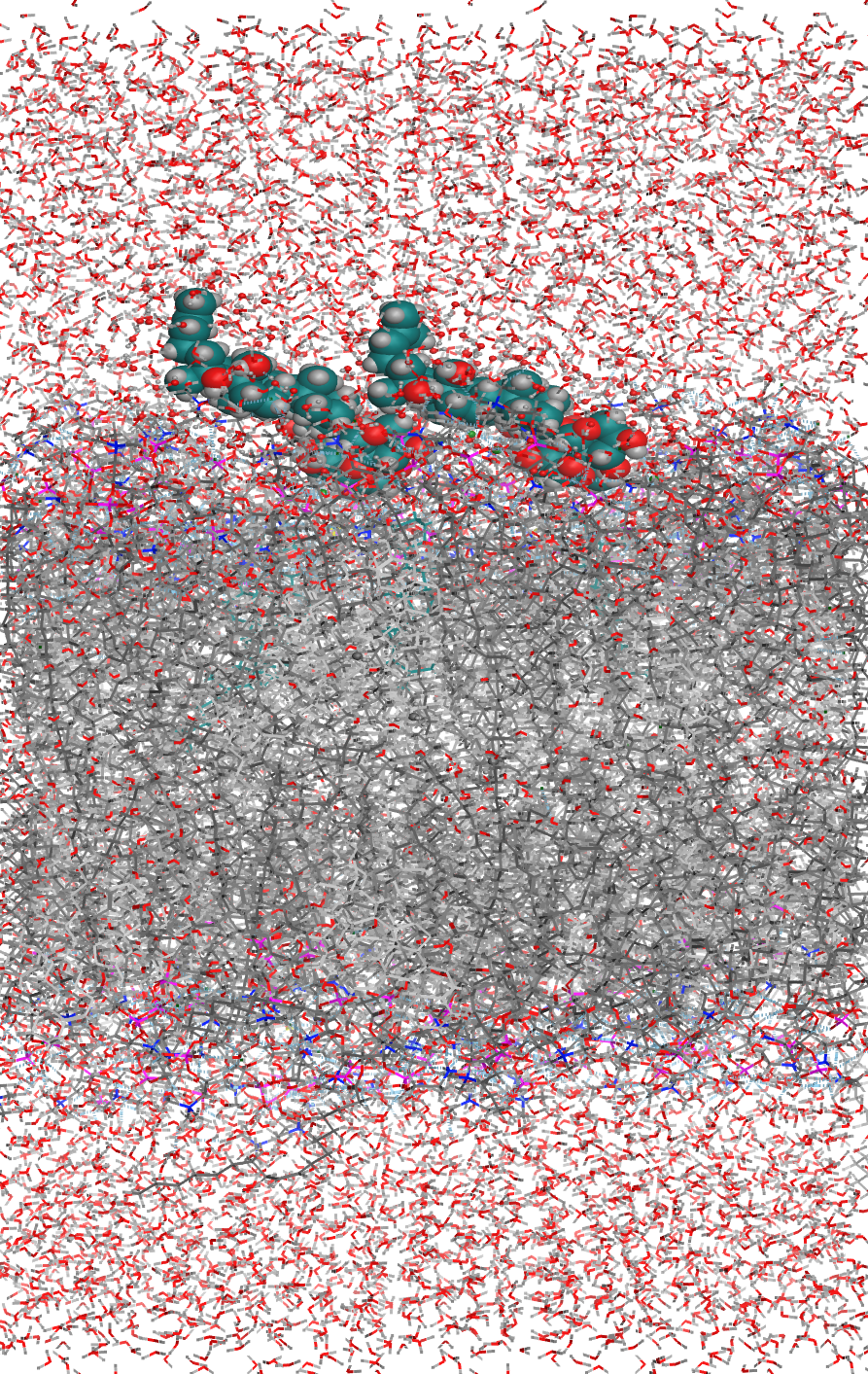

Supplement: Supplementary file 1 [file marinedrugs-19-00604-s001.zip › figure S2 Bcorp.jpg]

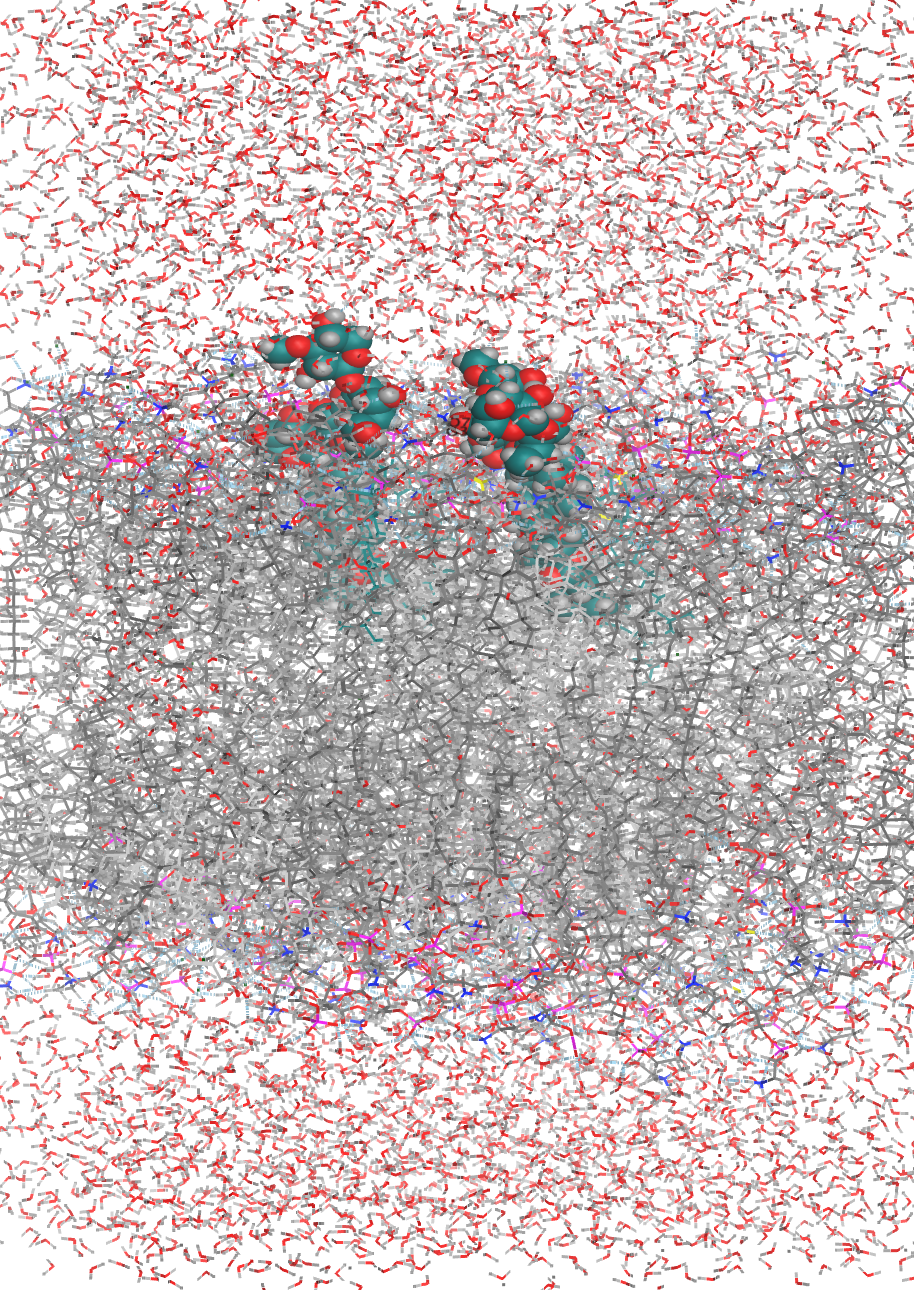

Supplement: Supplementary file 1 [file marinedrugs-19-00604-s001.zip › figure S2 Ccorp.jpg]

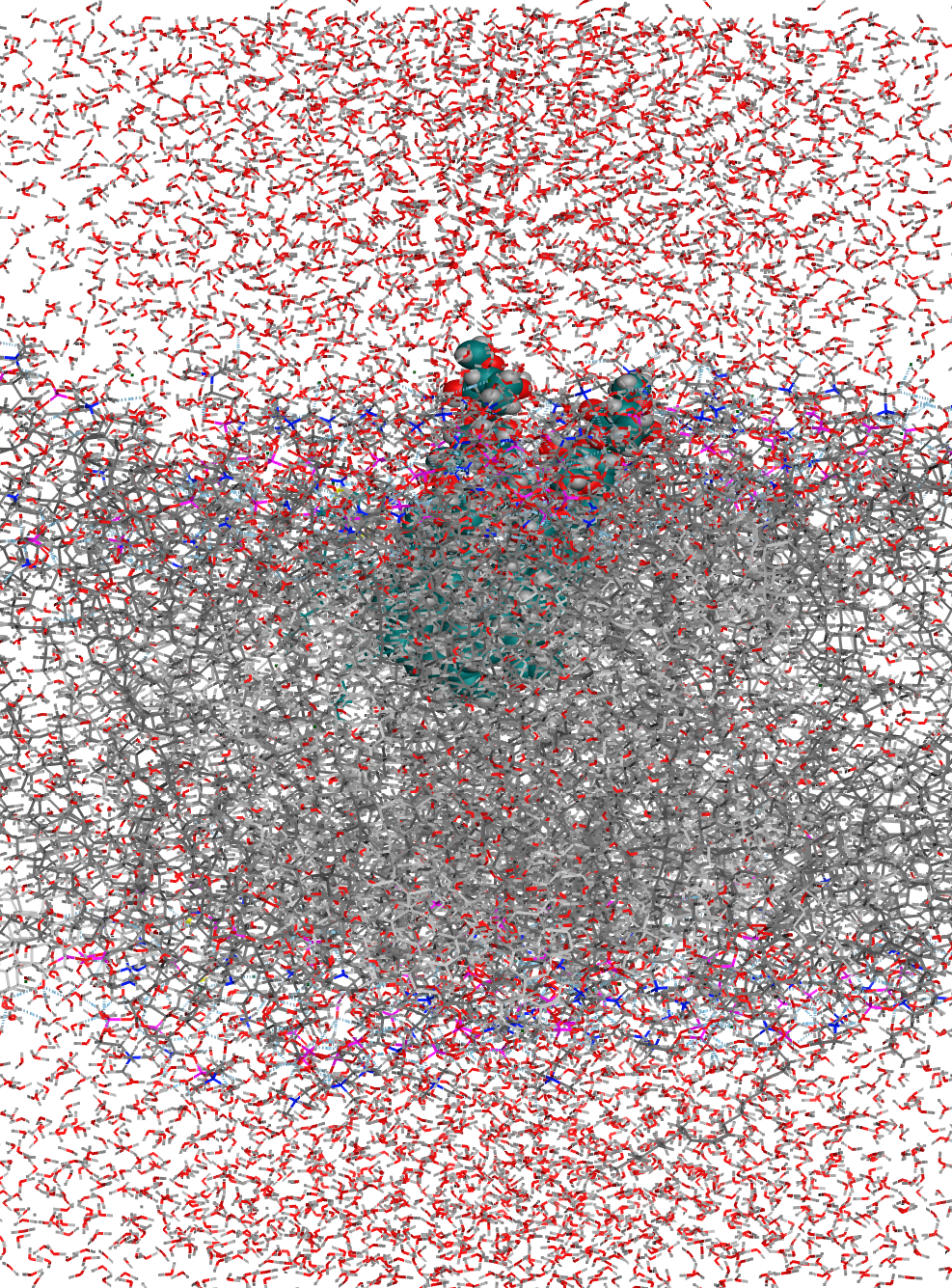

Supplement: Supplementary file 1 [file marinedrugs-19-00604-s001.zip › figure S2 D.jpg]
